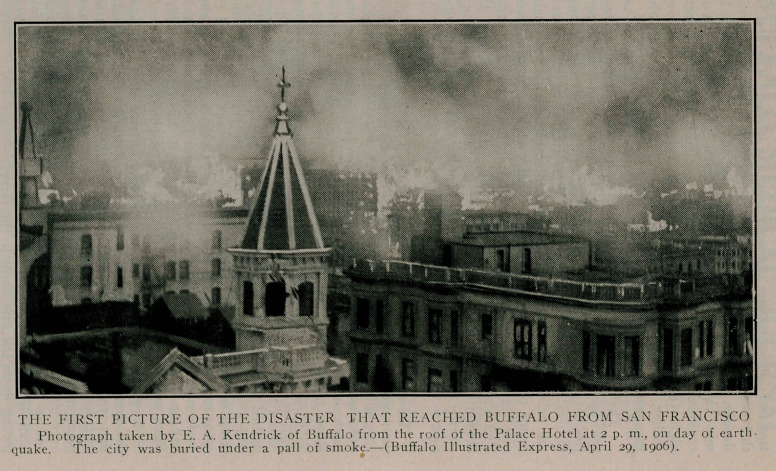# Thc San Francisco Calamity

**Published:** 1906-05

**Authors:** 


A Monthly Review of Medicine and Surgery.

EDITOR:

WILLIAM WARREN POTTER, M. D.

All communications, whether of a literary or business nature, books for review and exchanges, should be addressed to the editor 284 FRANKLIN ST., BUFFALO, N. Y.

VOL. LXI. MAY, 1906. NO. 10

### The San Francisco Calamity

The city of San Francisco was awakened on the morning of April 18, 1906, by one of the most remarkable seismic disturbances which the North American continent has experienced in modern times. The earthquake, which happened about 5.20 a. m., had scarcely ceased when fire broke out in three different localities, and for two days a conflagration raged which threatened to annihilate our fair Franciscan sister. Water mains were broken, reservoirs disabled, and the usually efficient modern means of fighting fire were practically put out of commission, leaving dynamite the only remaining agent with which to check the progress of the flames.

Whole blocks of buildings lining long streets and avenues were razed by the explosive before the mad march of destruction could be arrested. When the end had come it was found that upwards of 300 lives were lost, more than $200,000,000 of property had been destroyed and two-thirds of the great metropolis of the Pacific slope had been wiped out, rendering 300,000 people homeless. A great nation was in sympathetic mourning for this stricken people and within a week contributed more than $20,000,000 to the temporary relief of a suffering community. But above and beyond all, the San Franciscans themselves immediately arose to the occasion and began to reconstruct their almost ruined city. It is pleasant to help those who help themselves. In spite of the discouraging outlook, there can be no doubt that San Francisco will rebuild itself on a scale of unsurpassed splendor, and in five years scarcely a trace will be left to indicate the devastation of 1906.

It is far from our purpose to describe in detail the circumstances of this unparalleled disaster, but simply to give a synopsis of its leading features for the purpose of pointing out one or two facts of importance connected with the horror. The first is, that despite the liability to pestilence of which there was great danger, none has followed in the wake of the catastrophe. It must not be forgotten that sewers and drains have been put out of commission and that 200,000 people have been living for a fortnight (at this writing) in improvised camps and other temporary shelter. Only the exercise of great vigilance could avert the ravages of pestilence in the presence of these conditions.

The second great lesson impressed by a study of the calamitous environment is the part played by the army in maintaining order, relieving suffering, and establishing efficient sanitary regulations. General Frederick Funston showed himself equal to the occasion and with a calm judgment took possession of the city and established police regulations, which restored confidence to a stricken and demoralised people. The soldiers of the army, under General Funston's direction, were charged with a delicate task and all accounts agree that they performed it with a fidelity to duty worthy of all praise. We quote the words of an intelligent witness to the facts, taken from the *Buffalo Express,* April 27, 1906:

Edward A. Kendrick, secretary of the J. N. Matthews Company, got home yesterday from San Francisco, where he had been caught in the earthquake. He had been on the coast for a pleasure trip and had arrived in San Francisco a few hours before the earthquake. Mr. Kendrick and his traveling companion, Horace Reed, registered in the Berkshire Hotel, a frame structure that stood up under the shock, though it rocked in the most terrifying manner. Mr. Kendrick realised it was an earthquake and not a dream and dressed most expeditiously. He and his companion dashed out into the street in the gray dawn of the morning. There was a heavy fog and the people were pouring down the street in frantic terror. The two men went back to the hotel to assist two women relatives and then Mr. Kendrick went upon the roof to take photographs. He secured some good results, which will be shown in The Illustrated Express. Later, he and Mr. Reed walked down Market street for several blocks.

Fire now had broken out and on every corner they could see the firemen and apparatus vainly endeavoring to find hydrants that were not dry. The fires spread fast, but incredibly fast as was the terror that seized upon the city, greater by far was the promptness of the regular army men. The troops were rushed over from the Presidio by General Funston on the double quick and in no time, says Mr. Kendrick, the lads in khaki with full cartridge belts and rifles were guiding the frantic, terror-stricken throng down the safe roads to the ferry.

THE FIRST PICTURE OF THE DISASTER THAT REACHED BUFFALO FROM SAN FRANCISCO

Photograph taken by E. A. Kendrick of Buffalo from the roof of the Palace Hotel at 2 p. m., on day of earthquake. The city was buried under a pall of smoke.—(Buffalo Illustrated Express, April 29, 1906).

The troops were on hand so promptly that Mr. Kendrick, when he started to walk down Market street, had not gone four blocks before a regular army man brought him to a halt.

"No word of praise can ever do justice to those men of our own Thirteenth," said Mr. Kendrick. "They formed a thin line against which the fear-mad mob surged and broke away in groups with some confidence and some purpose. The great fear of more shocks was everywhere, but a sight of the regulars so cool and so unperturbed allayed the panic and the great throngs moved in the road indicated, much as sheep would move."

Whenever disaster or epidemic disease overtakes our people, it is the war department that first comes to the succor. The Secrtary of War orders tents and rations issued for temporary relief and sends troops to the scene to preserve order and maintain sanitary discipline. In the present instance Secretary Taft, on humanitarian grounds, felt compelled to exceed the authority conferred upon him by law and expend money at once that had been appropriated for other purposes. He thus became liable to impeachment but fell back into the arms of a generous congress for vindication.

This should not be in this land of peace and plenty. Some provision should be made whereby the Secretary of War may become possessed of an adequate emergency fund—say $3,000,000 or $5,000,000—to meet the exigencies of possible calamity. Moreover, congress should cease its niggardly course toward the army, and increase its numbers and enlarge its appropriations in order to adequately meet the necessities of this great right arm of civil government. The army is the friend of the people. It is our protector, defender, and sympathizer. It strikes down our foes in war, it folds us in its tender arms in peace, and it ministers unto us in affliction. All hail! then, to the Army of the United States!

---

The physicians of San Francisco in common with other citizens have suffered immense losses. They need instruments, books, and other equipment for their offices, and even money in many instances would be most acceptable. We will undertake to transmit any contributions that may be made from this region, to the San Francisco Medical Society, which seems much better than to send through a public relief bureau.

## Figures and Tables

**Figure f1:**